# The natural history of insomnia: predisposing, precipitating, coping, and perpetuating factors over the early developmental course of insomnia

**DOI:** 10.1093/sleep/zsab095

**Published:** 2021-04-13

**Authors:** Jason G Ellis, Michael L Perlis, Colin A Espie, Michael A Grandner, Célyne H Bastien, Nicola L Barclay, Ellemarije Altena, Maria Gardani

**Affiliations:** 1 Northumbria Sleep Research, Department of Psychology, Northumbria University, Newcastle upon Tyne, UK; 2 Department of Psychiatry, University of Pennsylvania, Philadelphia, PA; 3 Sleep and Circadian Neuroscience Institute (SCNi), University of Oxford, Oxford, UK; 4 Department of Psychiatry, Psychology and Medicine, University of Arizona, Tucson, AZ; 5 School of Psychology, Université Laval, Quebec, Canada; 6 CERVO Research Centre, Quebec, Canada; 7 School of Psychology, University of Glasgow, Glasgow, UK; 8 UMR 5287, Institut de Neurosciences Intégratives et Cognitives d’Aquitaine, Neuroimagerie et Cognition Humaine, CNRS, Université de Bordeaux, Bordeaux, France

**Keywords:** acute insomnia, sleep preoccupation, prevention, Spielman, depression

## Abstract

While there is an extensive literature on predisposing, precipitating, coping, and perpetuating factors in those with chronic insomnia, very little work has been undertaken to evaluate these factors over the early developmental course of insomnia. The present aim was to determine whether several hypothesized factors in each domain (predisposing, precipitating, coping, and perpetuating), assessed during an episode of acute insomnia (AI), are related to its persistence or remission to normal sleep. Participants comprised *n* = 140 people with AI and *n* = 737 normal sleepers (NS) recruited from the general public. Participants completed measures assessing predisposing characteristics (personality traits, arousal predisposition, and insomnia vulnerability), precipitating events and outcomes (life events, perceived stress, anxiety, and depression), coping styles (thought control strategies and coping styles), and perpetuating factors (sleep preoccupation, pre-sleep arousal, dysfunctional beliefs, and fatigue). Additionally, insomnia status (from AI at baseline to its persistence or natural remission [NR]) was assessed 1 month later (*n* = 129). Baseline differences between NS and individuals with AI were observed in each domain with increasing age, lower openness to experience and conscientiousness, higher insomnia severity, levels of anxiety, and affective sleep preoccupation significantly predicting AI status. Further, a previous episode of insomnia, higher depression scores, and affective sleep preoccupation scores significantly predicted its persistence, as opposed to its NR. Results are discussed with reference to the conceptualization of insomnia and how the findings may influence the design of preventative interventions to circumvent the transition from acute to chronic insomnia.

**Statement of Significance**

This study is the first of its kind to examine the role of predisposing, precipitating, coping, and perpetuating factors over the early developmental course of insomnia. Starting with a sample of individuals with acute insomnia (AI) and a group of normal sleepers (NS) the aims were to determine the predisposing, precipitating, coping, and perpetuating factors that differentiated and predicted group membership. Further, these factors were examined in the natural remission (NR) or its persistence. The findings add weight to Spielman’s 3P model of insomnia in terms of the relevance of these factors over the early developmental course of insomnia. Additionally, the findings are significant in terms of developing interventions both to manage AI and prevent chronic insomnia using a CBT-I format.

## Introduction

While there are a variety of etiologic theories about insomnia [1], the first and most well-known model is the Spielman or “3P” model [2, 3]. Spielman conceptualized insomnia along a trajectory from normal sleep to chronic insomnia via two transition points—acute (onset) and early (short-term) insomnia. Within this framework, Spielman suggested differing levels of influence from a range of predisposing, precipitating, and perpetuating factors. Predisposing factors (e.g. personality traits) run through the entire course of the disorder making some more vulnerable to insomnia than others. These predisposing factors are then compounded by a precipitating event (e.g. a stressor) that pushes the individual above an “insomnia threshold” with acute sleep disruption, or acute insomnia (AI), being the result. Over time, the impact of the stressor starts to diminish but still remains the main factor fuelling the insomnia (early insomnia) while perpetuating factors (e.g. learned negative associations, behaviors and cognitions which further inhibit the sleep process) are introduced. Finally, the impact of the stressor becomes negligible, but it is perpetuating factors that keep the individual over the insomnia threshold (chronic insomnia).

While this conceptualization has spawned a myriad of theories, models and empirical investigations related to the maintenance of insomnia, the “pre-chronic” (i.e. AI and early insomnia) stage has not been thoroughly examined, despite its potential as a target for preventative campaigns [4]. One issue, which most likely influenced the lack of research in this area, is that where Spielman discussed the course of insomnia, the temporality of each stage (i.e. when does AI become chronic insomnia) was never specified. Moreover, the duration for a diagnosis of chronic insomnia, and by definition AI, has changed several times and has not always been consistent between competing nosologies [5].

Significant amounts of cross-sectional, longitudinal, and experimental evidence provide support for Spielman’s conceptualization of insomnia, with a variety of predisposing, precipitating, coping, and perpetuating factors being shown to differentiate normal sleepers (NS) from those with insomnia (e.g. personality traits, stress, maladaptive coping styles) [6–8]. That said, the majority of this research has not: (1) compared NS against individuals in the AI phase, (2) used the current duration criteria to differentiate acute from chronic insomnia (i.e. 3 months), (3) employed measurement intervals which would afford an examination of the acute phase (e.g. most have annual follow-ups), and/or (4) used standardized questionnaires.

Considering Spielman’s conceptualization is a stress-diathesis model; however, speculations regarding specific predisposing, precipitating, coping, and perpetuating factors in the early developmental course of insomnia can be made. It would be reasonable to assume, for example, that predispositional characteristics that relate to increased physical and/or psychological arousal may be factors that increase the likelihood of sleep disruption in response to a stressor (e.g. neuroticism, insomnia vulnerability, and predispositional arousal). Furthermore, these predispositional characteristics are also likely to influence the switch from the stressor causing the insomnia to the insomnia becoming a stressor in itself (i.e. the transition from acute to chronic insomnia). A stressor, be it acute or chronic [5], which results in poorer mood and/or disruption to “typical” functioning, is also likely to be a factor in the initial onset of insomnia but become less relevant as the insomnia progresses into the chronic phase. Related to the stressor is the issue of coping. For the stressor to be perceived as such, as well as impacting upon mood and functioning, it could reasonably be assumed that coping resources (predispositional style and/or capacity) would either be limited or maladaptive. Considering that most of these factors are assumed to be trait like (e.g. personality and coping style) or only relevant for a short period of time (e.g. the stressor, coping capacity towards that stressor and its impact on mood), the most parsimonious way to examine the role of these factors over the developmental course of insomnia is by using the law of initial values.

The most analogous sample to that of AI,^1^ that has been studied, is subsyndromal insomnia, defined as presence of insomnia symptoms which together meet most but not all DSM-IV criteria (e.g. the individual meets criteria for complaint, adequacy of sleep opportunity, dysfunction and duration but not frequency). One study compared NS and individuals with subsyndromal insomnia on several predisposing, precipitating, and coping variables [9, 10]. The findings suggest that individuals with subsyndromal insomnia were older and reported higher levels of depression, anxiety, and stress, greater scores on measures of arousal predisposition, neuroticism, and maladaptive coping (emotion or avoidance focused), and lower scores on extraversion, compared to NS. Moreover, an increased vulnerability to stress-related insomnia, as measured by the Ford Insomnia Response to Stress Test (FIRST) [11], has been shown to predict new onset of both subsyndromal insomnia and chronic insomnia [12, 13]. Finally, one study has examined differences in thought control strategies between individuals with AI (defined as having insomnia for less than six months–ICSD-2 Criteria) against those with chronic insomnia [14]. They found that while both conditions were associated with worry, as a thought control strategy, AI was also characterized by punishment or self-blame. As such, there is some evidence, albeit cautiously due to these definitional differences, that specific predisposing, precipitating and coping factors may be important during the pre-chronic phase. There is also evidence, albeit limited, for the role of sleep preoccupation during AI, with one study demonstrating no differences between individuals with AI compared to chronic insomnia [15]. Although not examined within the context of subsyndromal or AI, it would also seem pertinent to examine additional perpetuating factors during this early stage of insomnia as (1) Spielman suggests their influence begins during the acute phase, (2) the transition point between acute and chronic insomnia is still unknown and (3) the considerable evidence for the role of perpetuating factors in chronic insomnia and its management [16, 17]. For those reasons, dimensions of pre-sleep arousal, dysfunctional beliefs, fatigue and sleep preoccupation were included in the study.

The aims of the present study include the following: (1) determine which predisposing, precipitating, coping, and perpetuating factors differentiate NS from those with AI and (2) determine which predisposing, precipitating, coping, and perpetuating factors predict whether an individual will transition from AI to natural remission (NR) or its persistence. It was hypothesized, in line with previous research, that specific predisposing (neuroticism, extraversion, arousal predisposition, and insomnia vulnerability), precipitating (perceived stress, anxiety, and depression), coping (coping styles and the thought control strategies of worry and punishment) and perpetuating (dysfunctional beliefs, fatigue, pre-sleep arousal and sleep preoccupation) factors would differentiate NS from individuals with AI. Further, it was hypothesized that factors in each domain would also significantly predict who would naturally remit from AI, compared to those whose insomnia would persist, though there were no specific hypotheses regarding which factors would demonstrate significant relationships in this regard.

## Methods

### Participants and procedure

Participants were recruited from the North of the United Kingdom using a variety of advertising methods—posters displayed in community settings (e.g. churches, libraries and universities), radio adverts, newspaper adverts, and email alerts to employees at various industries and at universities, asking for normally sleeping volunteers and individuals who were currently losing sleep due to stress^2^ to contact the research group. Upon calling, participants were told about the inclusion criteria (e.g. age range of 18–59 years, a normal sleeper or individual with insomnia for less than 3 months) and exclusion criteria (i.e. no other sleep disorder or currently receiving support for their sleep from a healthcare professional—including sleep medication use).

If eligible and interested, participants then completed informed consent before a semi-structured interview about their sleep. The interview consisted of three sections: (1) confirming eligibility on the inclusion and exclusion criteria (including 17 questions covering other sleep disorders according to the ICSD-2, for exclusion purposes, and two questions about treatment-seeking), (2) determining insomnia or normal sleeper status using a diagnostic algorithm (nine questions covering DSM-5 criteria for Insomnia Disorder and normal sleeper definitions), and (3) informing participants about the study in terms of how much input and effort was required. AI was defined from the algorithm as meeting all DSM-5 criteria for Insomnia Disorder except the duration criterion (i.e. they had to report insomnia for less than 3 months) and those that reported a prior episode of insomnia were included in the study. NS were defined on the basis of reporting being satisfied with their sleep, reporting no difficulties with sleep onset or maintenance or daytime sleepiness, and not taking any medication that could interfere with their sleep (from the diagnostic algorithm). If a potential participant reported a case of chronic insomnia (i.e. more than 3 months duration) or any other sleep disorder, they were thanked for their time and not enrolled in the study. If eligible, participants were then invited to take part in an online survey with four assessment points (baseline entry, 1 month post entry, 3 months post entry, and 6 months post entry). Those who agreed to take part were sent a link to the online survey containing an additional informed consent front page that had to be completed before they could access the survey. Email reminders were sent if a participant did not complete a phase of the survey 3 days after it was due to be completed. A maximum of three reminders were sent before concluding that a participant had withdrawn. On completion of the study participants were thanked for their time and debriefed. Recruitment was coordinated from two universities in the United Kingdom and ethical approval was sought and obtained at both sites.

### Measures

#### Sleep measures

The Insomnia Severity Index (ISI; [18, 19]) is a 7-item scale that examines the presence of insomnia symptoms over the previous 2 weeks on a five-point Likert scale (0 = None–4 = Severe). Scores are summed to provide a range between 0 and 28, with higher scores indicating higher symptom severity. The Pittsburgh Sleep Quality Index (PSQI; [20]) is a 19-item scale that assesses the presence and significance of sleep disturbances over the previous month. It comprises seven subscales which are rated on a scale of 0–3 to provide an overall range (Global Pittsburgh Sleep Quality Index) between 0 and 21, with higher scores indicating poorer sleep.

#### Predisposing measures

The NEO Five-Factor Inventory (NEO-FFI [21]) is a 60-item measure of trait personality and comprises five dimensions (neuroticism, extraversion, openness to experience, conscientiousness and agreeableness) with 12 items each. Each item is rated on a five-point Likert type scale (1 = Strongly agree–5 = Strongly disagree) and summed. Scores range from 1 to 60 on each dimension with higher scores indicating higher levels of that specific trait. The Arousal Predisposition Scale (APS; [22]) is a 12-item scale that measures individual differences in arousability. It comprises of a five-point Likert scale (0 = Never to 5 = Always) and scores are summed, after reversed scoring of one item, to provide a range between 0–60, with higher scores indicating a higher trait arousability. The FIRST [11] is a 9-item measure of vulnerability to sleep disruption in response to a stressor. It is scored on a four-point Likert scale (1 = Never to 4 = Always) and responses are summed to provide a range between 9 and 36. Higher scores indicate increased vulnerability. There are no timelines associated with the NEO-FFI, APS or FIRST.

#### Precipitant measures

The Hospital Anxiety and Depression Scale (HADS; [23]) is a 14-item scale that assesses levels of anxiety (seven items) and depression (seven items) over the previous month. Responses are recorded on a five-point Likert scale (e.g. 0 = Not at all to 5 = Definitely) with differing response formats. Scores are summed, after reversal of some items, to provide a range between 0 and 21 on each domain (anxiety and depression), with higher scores indicating a higher severity of symptoms. The Perceived Stress Scale (PSS; [24]) is a 10-item scale that measures stress perception over the previous month on a five-point Likert scale (0 = Never to 5 = Very often). Scores are summed, after reversal of some items, to provide a range between 0 and 40, with higher scores indicating higher levels of perceived stress. The Social Readjustment Rating Scale (SRRS; [25]) is a scale that contains 43 stressful life events (e.g. divorce) each of which are given a weighting. Participants indicate which events they have experienced over the previous year and the weighted scores are summed. Scores range from 0 to 1467 with higher scores indicating an increased susceptibility to stress-related illness.

#### Coping measures

The Brief COPE (Carver [26]) is a 28-item measure of coping in response to a stressor. Although it has 14 subscales (self-distraction, active coping, denial, substance use, use of emotional support, use of instrumental support, behavioral disengagement, venting, positive reframing, planning, humor, acceptance, religion, and self-blame), these can be condensed into two subscales (adaptive/maladaptive coping) [27] that are rated on a four-point Likert scale (1 = I haven’t been doing this a lot–4 = I have been doing this a lot). Scores on the adaptive subscale range from 8 to 16 and 6–12 on the maladaptive subscale with higher scores indicating more use of that coping style. The Thought Control Questionnaire (TCQ [28]) is a 30-item scale that assesses the strategies employed to deal with unwanted intrusive thoughts. There are five subscales (distraction, social control, worry, punishment and reappraisal) with six items in each which participants rate on a four-point scale (1 = Never–4 = Always). Scores on each subscale range from 6 to 24 with higher scores indicating more use of that thought control strategy. The summed score is regarded as the final outcome score. There is no timelines associated with the Brief COPE or TCQ.

#### Perpetuating measures

The Dysfunctional Beliefs and Attitudes to Sleep (DBAS-16 [29]) is a 16-item scale that assesses dysfunctional beliefs about the causes and consequences of insomnia. Each item is rated on a 100 mm visual analog scale and a mean score (0–100—with higher scores indicating higher levels of sleep-related dysfunctional thinking) are calculated. The Flinders Fatigue Scale (FFS; [30]) is a 7-item scale that asks about the frequency and timing experiencing symptoms of fatigue over the previous 2 weeks and was constructed within the context of insomnia. Scores are derived on each item separately and are then summed to provide an overall scale between 0 and 31, with higher scores indicating increased fatigue. The Pre-Sleep Arousal Scale (PSAS; [31]) is a 16-item scale that measures levels of arousal at bedtime. There are two subscales (somatic arousal and cognitive arousal), each with 8-items. All items are scored on a five-point Likert scale (1 = Not at all to 5 = extremely) and scores are summed, providing a range between 8 and 40 for each subscale with higher scores indicating higher levels of pre-sleep arousal. The Sleep Preoccupation Scale (SPS; [32]) is a 22-item scale that measures levels of daytime preoccupation about sleep over the previous month. Each item is rated on a seven-point Likert scale (0-Never to 6-All the Time). There are two subscales—cognitive and behavioral preoccupation—which relates to perceived impairments and behavioral coping strategies in response to poor sleep (14 items range 0–84) and affective preoccupation which relates to worry, concern, and rumination over poor sleep (8 items range 0–48) and higher scores indicate higher levels of sleep preoccupation.

### Insomnia status at follow-up

The one-month follow-up data on the ISI was used to determine whether the participant had naturally remitted or whether the insomnia persisted. As per Morin et al. [33], an ISI score *>*10, with no reported change in sleep status from baseline (yes/no response—self-reported item in the online survey), indicated that the participants insomnia had persisted insomnia (PI) and an ISI score <10 with a reported improvement in their sleep since baseline (yes/no response) and no use of sleep medication (both self-reported) indicated NR.

### Data analysis

Between-group differences (AI vs. normal sleeper) were examined using independent *t*-tests and Chi-square tests. Multi-comparisons Bonferroni post-hoc corrections were applied to these analyses (.05/26 = *p* < .002). Additionally, due to the unequal group sizes, unequal size t-values are reported. In all other analyses *p* < .05 was used. To examine the predictors of status (AI vs. normal sleeper) and change in status (i.e. from AI to either its natural remission or its persistence) a series of stepwise hierarchical logistic regressions were conducted. The steps of the regression were chosen on the basis of Spielman’s’ model (e.g. block 1: predisposing factors (including age), block 2: precipitating events and outcomes, block 3: coping styles and block 4: perpetuating factors). The choice of which variables to include was done on the basis of the *t*-test results (i.e. which variables differentiate NS from individuals with AI). The rationale for this was that these comparisons would provide the broadest range of differentiating factors (i.e. high sensitivity) which would feed into the regressions to provide more specific information on the predictive power of those factors (high specificity). For each analysis, parametric assumptions specific to each test (e.g. normality, outliers, and muticollinearity) were examined and are reported where necessary. Missing data were treated by mean substitution if less than 5% of a measure was missing or casewise deletion if more than 5% of a measure was missing. Although there are cut-off scores for most, if not all, the measures used in the study, a choice was made to keep all the variables continuous as dichotomizing reduces power, especially within the context of regression analyses.

## Results

**Figure 1.**
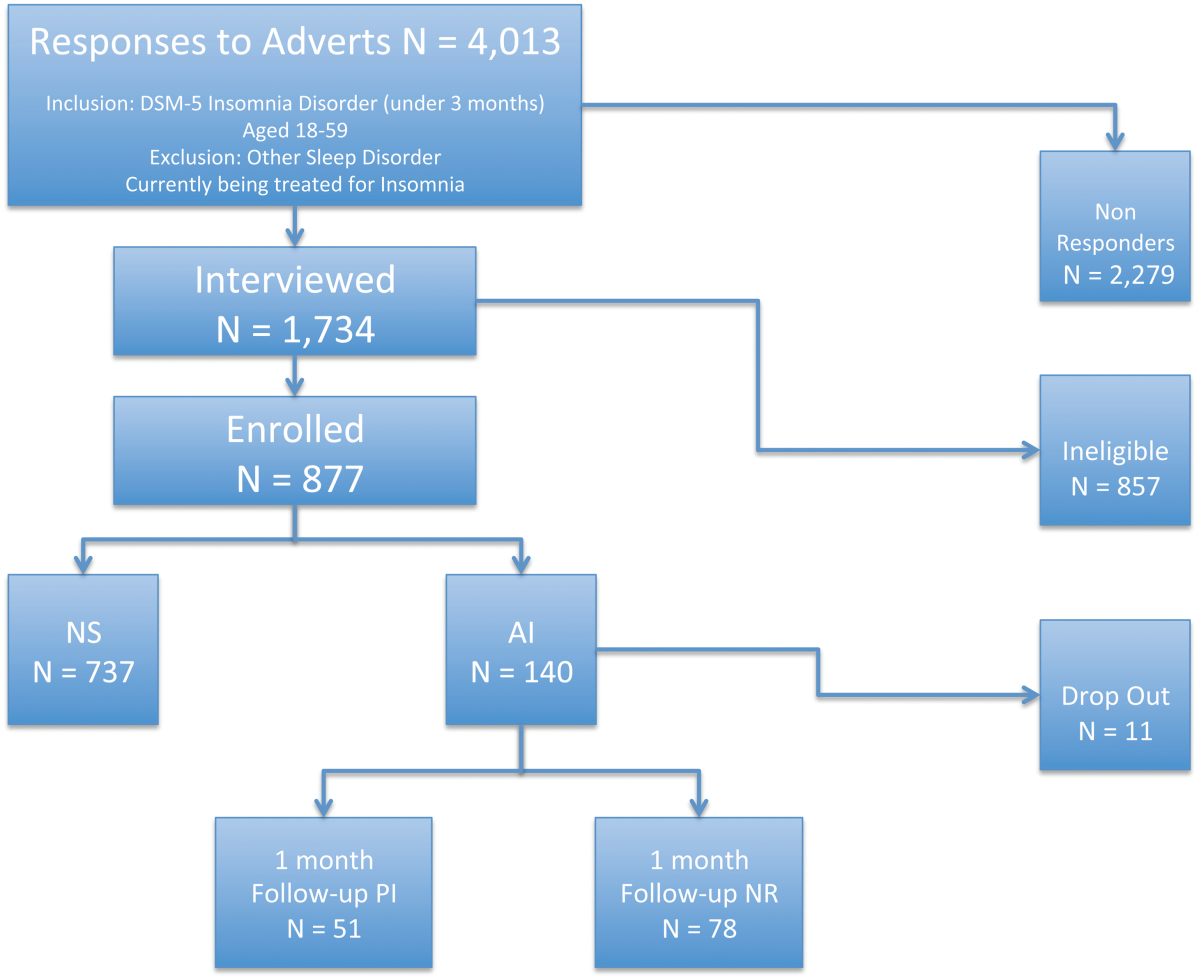
Participant flow.

Of the 4037 enquiries received, 877 individuals (737 NS and 140 individuals with AI) were enrolled on to the study (see Figure 1 for participant flow). Of those, 644 (73%) were females and 233 (27%) were males and the mean age of the sample was 29.33 ± 11.57. Of note, 366 individuals were excluded from the study due to reporting chronic insomnia, another sleep disorder or use of sleep medication.

Those with AI were significantly older (Mean age 31.91 ± 12.47 years) than those reporting normal sleep (Mean age 26.74 ± 10.67 years) (*t*(875) = 4.59, *p* = .001). There were no differences between those with AI and NS on any other demographic variables (i.e. sex, education level, marital status, and ethnicity—all at *p* > .05) (see Table 1 for demographics by group). Of those with AI (*n* = 140), 81 (57.86%) reported this insomnia event as their first-ever-episode, 59 (42.14%) reported it as a recurrent episode. The overall mean duration of their insomnia was 8.02 ± .95 weeks (range 2–12 weeks). As would be expected, there were significant differences between AI and NS on measures of sleep disturbance; both on the PSQI (*t*(875) = 17.83, *p* = .001—Mean AI = 13.51 ± 5.45, Mean NS = 4.56 ± 3.71) and ISI (*t*(875) = 18.63, *p* = .001—Mean AI = 10.82 ± 3.12, Mean NS = 5.87 ± 2.38), with those with AI reporting higher levels of sleep disturbance than NS (Table 2).

**Table 1.** Sample demographics by group

	Normal sleepers (*N* = 737)	Acute insomnia (*N* = 140)
Sex (Female)	548 (74.4%)	96 (67.9%)
Martital status		
Married/Cohabiting	367 (49.8%)	75 (53.57%)
Single	348 (47.22%)	49 (35%)
Divorced/Separated	20 (2.71%)	14 (10%)
Widowed	2 (0.27%)	2 (1.43%)
Ethnicity		
White	694 (94.17%)	126 (90%)
Black	9 (1.22%)	7 (4.5%)
Asian	24 (3.26%)	4 (2.86)
Other	10 (1.36%)	3 (2.14%)
Education Level		
School	410 (55.63%)	36 (25.71%)
College	83 (11.26%)	47 (33.57%)
Degree	148 (20.08%)	27 (19.29%)
Higher Degree	68 (9.23%)	20 (14.29%)
Doctorate	28 (3.8%)	10 (7.14%)

**Table 2.** Baseline differences between normal sleepers and individuals with acute insomnia

	Normal sleepers (*N* = 737)		Individuals with acute insomnia (*N* = 140)		*t*	*p*
	Mean	SD	Mean	SD		
Sleep/insomnia symptoms						
ISI—Insomnia Severity	4.56	3.71	13.51	5.45	18.63	.001*
PSQI—Sleep Disturbance	5.87	2.38	10.82	3.12	17.83	.001*
Predisposing factors						
NEO—Neuroticism	37.52	9.81	43.71	11.63	5.91	.001*
NEO—Extraversion	49.28	7.48	45.73	9.46	−4.2	.001*
NEO—Openness	46.07	7.27	48.52	8.2	3.23	.001*
NEO—Agreeableness	53.39	6.51	52.14	7.32	−1.88	.06
NEO—Conscientiousness	52.39	7.71	48.95	9.27	−4.13	.001*
FIRST—Insomnia Vulnerability	20.22	5.17	23.86	5.55	7.17	.001*
APS—Arousal Predisposition	31.56	7.1	34.56	8.1	4.1	.001*
Precipitating outcomes						
Life Events	155.11	96.42	180.95	99.98	2.82	.004
PSS—Perceived Stress	37.61	7.02	43.4	7.45	8.51	.001*
HADS—Anxiety	6.29	3.9	9.83	4.17	9.3	.001*
HADS—Depression	4.46	2.76	7.14	3.41	8.8	.001*
Coping strategies						
TCQ—Worry	9.69	2.83	10.77	2.91	4.06	.001*
TCQ—Distraction	14.95	2.89	14.37	3.32	−1.92	.056
TCQ—Punishment	9.46	2.39	10.37	2.86	3.52	.001*
TCQ—Reappraisal	13.14	3.42	13.73	2.95	2.11	.035
TCQ—Social Control	12.97	4.23	12.28	3.99	−1.85	.065
BRIEF COPE—Adaptive	15.55	5.51	17.05	5.49	−2.96	.003
BRIEF COPE—Maladaptive	13.09	4.72	13.69	4.48	1.45	.15
Perpetuating factors						
FFS—Fatigue	11.03	5.2	17.45	5.6	12.58	.001*
DBAS-16—Dysfunctional Beliefs	58.71	22.46	82.25	26.36	9.91	.001*
SPS-CB—Cog/Behav Sleep Preoccupation	45.34	14.08	54.32	13.89	7	.001*
SPS-A—Affective Sleep Preoccupation	14.27	5.57	24.66	8.64	13.7	.001*
PSAS-S—Somatic Pre-Sleep Arousal	10.58	3.52	13.45	5.21	6.24	.001*
PSAS-C—Cognitive Pre-Sleep Arousal	13.24	4.5	16.48	4.78	7.4	.001*

ISI, insomnia severity index; PSQI, Pittsburgh Sleep Quality Index; NEO, five factor inventory; FIRST, ford insomnia response to stress test; APS, arousal predisposition scale; PSS, perceived stress scale; HADS, Hospital Anxiety and Depression Scale; TCQ, thought control questionnaire; BRIEF COPE, brief cope; FFS, fliders fatigue scale; DBAS 16, dysfunctional beliefs and attitudes to sleep scale (16 Item); SPS, sleep preoccupation scale; PSAS, pre-sleep arousal scale.

### Differences in predisposing characteristics

There were significant differences between AI and NS on arousal predisposition scores (APS) (*t*(875) = 4.48, *p* = .001), insomnia vulnerability (FIRST) scores (*t*(875) = 7.52, *p* = .001) and the personality dimensions of Neuroticism (*t*(875) = 6.64, *p* = .001), Extraversion (*t*(875) = −4.92, *p* = .001), Conscientiousness (*t*(875) = −4.68, *p* = .001) and Openness to Experience (*t*(875) = 3.58, *p* = .001). All variables (i.e. FIRST, APS, Neuroticism and Openness to Experience) with the exception of Extraversion and Conscientiousness were more highly endorsed by AI compared to NS (Table 2).

### Differences in precipitating characteristics

Those with AI reported higher levels of stress in terms of perceived stress (*t*(875) = 8.51, *p* = .001) and on both dimensions of the HADS (anxiety—*t*(875) = 9.74, *p* = .001; depression—*t*(875) = 10.14, *p* = .001) (Table 2). AI’s scored higher on the life events scale, compared to NS, this was not significant after the Bonferroni correction.

### Differences in coping characteristics

In terms of coping styles, no variables significantly differentiated AI from NS. For thought control strategies, as previously shown, individuals with AI scored higher than NS on both the worry (*t*(875) = 4.13, *p* = .001) and punishment (*t*(875) = 3.98, *p* = .001) dimensions of the TCQ.

### Differences in perpetuating factors

Levels of fatigue (*t*(875) = 13.24, *p* = .001), dysfunctional beliefs (*t*(875) = 11.05, *p* = .001), pre-sleep arousal (both the somatic and cognitive dimensions (*t*(875) = 8.08, *p* = .001 and *t*(875) = 7.72, *p* = .001, respectively) and levels of sleep preoccupation (both the cognitive and behavioral and affective dimensions (*t*(875) = 6.94, *p* = .001 and *t*(875) = 18.29, *p* = .001, respectively) differentiated AI from NS in the expected direction (i.e. higher levels of fatigue, more dysfunctional beliefs, higher pre-sleep arousal and higher levels of sleep preoccupation in the AI group compared to the NS group).

### Predictors of group membership (AI vs. NS)

A stepwise hierarchical logistic regression was undertaken with only those variables that significantly differed between those with AI and NS included as covariates (except PSQI scores due to multicollinearity). There were five blocks in the model (1= age, 2= neuroticism, extraversion, conscientiousness, openness to experience, FIRST scores and APS scores, 3= anxiety, depression and perceived stress, 4 = worry and punishment, and 5= dysfunctional beliefs, fatigue, sleep preoccupation and pre-sleep arousal scores). Prediction accuracy without covariates was 84% (100% for NS and 0% for AI status). This increased to 90.3% (96.7% NS and 56.4% for AI status) with the inclusion of the variables. The model, with the included variables, was significant (χ ^2^ = 360.27, *df* = 14, *p* = .001) and showed a good fit (χ ^2^ = 4.1, *df* = 7, *p* = .85). Although 57.6% of the variance was explained with the inclusion of the variables, only age (β = .03), ISI scores (β = .31), openness to experience scores (β = .04), conscientiousness scores (β = −.03), anxiety scores (β = .11), and affective sleep preoccupation (β = .07) significantly predicted group membership. In this case, being older and reporting higher insomnia severity, openness to experience, anxiety, and affective sleep preoccupation were predictive of having AI whereas higher conscientiousness scores significantly predicted being a normal sleeper.

### Demographic differences in group membership (NR vs. PI)

At the one-month follow-up, 129 of the 140 (92.14%) participants completed the ISI and answered the questions regarding changes in insomnia status. Of those, 78 (60.5%) were now classified as Natural Remitters (NR; an ISI score <10 and a reported improvement in their sleep since baseline) and 51 (39.5%) were classified as having an on-going Persistence of their Insomnia (PI; an ISI score *>*10 and no reported change in sleep status from baseline). Of the 129 participants, 41 (31.78%) were males and 88 (68.22%) were females and 75 (58.14%) had reported this as a first episode at baseline and 54 (41.86%) as a recurrent episode at baseline. There were no differences in age between those who had remitted (Mean age 30.51 ± 11.65 years) and those whose insomnia persisted (Mean age 32.57 ± 13.01 years) (*t*(127) = .94, *p* = .48). There were also no differences on any other demographic variables (all at *p* > .05), nor were there between-group differences on episode status— first episode or recurrent episode (χ ^2^ = 2.52, *df* = 1, *p* = .11).

### Differences between those who remit and those whose insomnia persisted

A series of *t*-tests showed that baseline scores on the ISI (*t*(127) = 4.4, *p* = .001) significantly differentiated the groups, with those whose insomnia persisted demonstrating higher symptom severity, at baseline, compared to those who remitted (Table 3). Although scores on the depression dimension of the HADS, DBAS, FFS, and affective dimension of the SPS differed between NR and PI’s (with PI’s having higher baseline scores in each domain), these were nonsignificant after the Bonferroni correction.

**Table 3.** Baseline differences between those who naturally remit and those whose insomnia persists

	Natural remitters (*N* = 78)		Persistent insomnia (*N* = 51)		*t*	*p*
	Mean	SD	Mean	SD		
Sleep/insomnia symptoms						
ISI—Insomnia Severity at Baseline	11.94	5.81	16.04	4.02	4.4	.001*
PSQI—Sleep Disturbance	10.3	3.09	11.75	3.21	2.56	.012
Predisposing factors						
NEO—Neuroticism	42.4	11.4	46	11.4	1.76	.082
NEO—Extraversion	46.13	9	45.18	10.36	−0.55	.582
NEO—Openness	48	8.08	48.55	8.73	0.37	.713
NEO—Agreeableness	51.9	6.58	51.53	8.15	−0.29	.775
NEO—Conscientiousness	48.41	8.87	49.33	9.38	0.57	.571
FIRST—Insomnia Vulnerability	23.5	5.78	24.82	5.1	1.33	.186
APS—Arousal Predisposition	34.01	8.2	35.98	7.29	1.39	.167
Precipitating outcomes						
Life Events	181.86	103.66	181.04	97.99	−0.05	.96
PSS—Perceived Stress	42.77	7.71	44.79	7.19	1.5	.136
HADS—Anxiety	9.53	4.53	10.55	3.66	1.38	.174
HADS—Depression	6.69	3.21	8.2	3.6	2.52	.013
Coping strategies						
TCQ—Worry	10.56	2.86	11.22	3.02	1.24	.218
TCQ—Distraction	14.54	3.29	14.16	3.35	−0.65	.518
TCQ—Punishment	10.32	2.8	10.45	2.86	0.26	.792
TCQ—Reappraisal	13.64	2.83	14.16	3.28	0.95	.345
TCQ—Social Control	12.12	3.73	12.7	4.43	0.81	.42
BRIEF COPE—Adaptive	16.67	5	17.88	6.36	−1.21	.23
BRIEF COPE—Maladaptive	13.52	4.72	14.06	4.5	−0.65	.52
Perpetuating factors						
FFS—Fatigue	16.41	5.94	19.12	4.51	−2.77	.006
DBAS-16—Dysfunctional Beliefs	79.04	26.03	89.72	26.58	−2.26	.026
SPS-CB—Cog/Behav Sleep Preoccupation	54.93	14.74	54.17	12.73	0.3	.76
SPS-A—Affective SleepPreoccupation	23.31	8.74	27.56	8.3	−2.76	.007
PSAS-S—Somatic Pre-Sleep Arousal	13.39	5.61	13.84	4.87	−0.48	.64
PSAS-C—Cognitive Pre-Sleep Arousal	16.14	4.84	17.16	4.41	−1.21	.23

ISI, insomnia severity index; PSQI, Pittsburgh Sleep Quality Index; NEO, five factor inventory; FIRST, ford insomnia response to stress test; APS, arousal predisposition scale; PSS, perceived stress scale; HADS, Hospital Anxiety and Depression Scale; TCQ, thought control questionnaire; BRIEF COPE, brief cope; FFS, fliders fatigue scale; DBAS 16, dysfunctional beliefs and attitudes to sleep scale (16 Item); SPS, sleep preoccupation scale; PSAS, pre-sleep arousal scale.

### Predictors of group membership (NR vs. PI)

A stepwise hierarchical logistic regression was conducted with all the significant variables that differentiated individuals with AI from NS, at baseline, included as covariates. Age, sex and episode history were also included in the model. There were five blocks in the model (1 = age sex and episode history, 2= neuroticism, extraversion, conscientiousness, openness to experience, FIRST scores and APS scores, 3 = anxiety, depression and perceived stress, 4 = worry and punishment, and 5 = dysfunctional beliefs, fatigue, sleep preoccupation and pre-sleep arousal scores). Prediction accuracy without covariates was 60.5% (100% for NR and 0% for PI status). This increased to 76% (85.9% NR and 60.8% for PI status) with the inclusion of the variables. The model with the included variable was significant (χ ^2^ = 40.38, *df* = 20, *p* = .004) and showed good fit (χ ^2^ = 9.17, *df* = 8, *p* = .33). The significant predictors of group status were episode history (β = 1.12), depression (β = .3); cognitive and behavioral sleep preoccupation (β = −.1) and affective sleep preoccupation scores (β = .1). In this case, reporting a previous episode of insomnia, higher depression scores and higher affective sleep preoccupation at baseline were predictive of those whose insomnia persisted whereas higher cognitive and behavioral sleep preoccupation was predictive of remission.

## Discussion

The present study aimed to examine predisposing, precipitating, coping, and perpetuating factors over the developmental course of insomnia. It was expected that factors in each domain would differentiate those with AI from NS. Also, it was expected that those same factors that differentiated AI from normal sleep would also predict group membership (NS vs. AI). Further, the study aimed to explore which factors would predict those who would remit from their AI from those whose insomnia would persist. The findings suggest that although variables in each domain differentiated NS from those with AI, only age, insomnia severity, the personality dimensions of openness to experience and conscientiousness, anxiety, and affective sleep preoccupation significantly predicted group membership. Moreover, only baseline depression levels and cognitive and behavioral and affective sleep preoccupation levels predicted who would naturally remit against those whose insomnia would persist.

Considering the previous literature, it was unsurprising that the severity of the initial sleep complaint (both on the ISI and PSQI) and the predisposing characteristics of neuroticism, extraversion, insomnia vulnerability, and arousal predisposition differentiated those with AI from NS [9–12]. Certainly from one prospective study, the initial severity of sleep complaints [34] was demonstrated to be a significant predictor for the development of insomnia, following hospitalization. It was also expected that the precipitating factors—anxiety, depression and perceived stress—and the thought control strategies of worry and punishment also differentiated groups. These latter findings mirror previous studies in that AI is associated with high levels of distress and more maladaptive thought control strategies [14, 35].

The finding that the “openness to experience” dimension of the NEO-FFI was more highly endorsed by those with AI than NS is interesting. Higher levels of openness are associated with an active imagination, aesthetic sensitivity, attentiveness to inner feelings, preference for variety and intellectual curiosity, none of which have previously been associated with insomnia, either positively or negatively. The other finding with regard to predisposing factors was that conscientiousness was more highly endorsed by NS compared to those reporting AI, again a characteristic not usually associated with insomnia. Whether these factors indirectly influence the development of AI following an initial period of sleep disruption in terms of how an individual responds to the initial sleep disturbance should be examined further.

It was interesting that scores on the Life Events Scale did not differentiate those with AI from NS, especially when considering the majority of individuals with insomnia (78.8%) can recall a specific event that precipitated their insomnia, on average, 11 years later [7]. That being said, this finding matches one study, using the same scale, which found no differences between individuals with insomnia and NS on the number of life-events experienced, but rather how stressful they felt the event was [36].

The finding that the included variables added to the prediction accuracy—adds support to Spielman’s model in that the acute phase of insomnia is characterized by a combination of predisposing, precipitating, coping, and perpetuating factors. That said, the finding that anxiety scores were a significant predictor of group membership whereas depression was not is curious. One speculation, based on previous research, is that anxiety characterizes the onset and acute phase of insomnia, whereas depression may be a consequence of insomnia that builds over time but is not central to its initial onset [37–39].

The final set of findings, that no predisposing, precipitating (except depression) or coping factors predicted the transition from AI to its persistence is interesting. Even more so, considering at least 25% of the sample would have still been classed as having AI according to DSM-5 criteria (i.e. less than three months) at the follow-up point. This adds support to Spielman’s model in that predisposing and precipitating factors become less relevant, if of any relevance at all, when the insomnia becomes chronic. What this finding, or lack thereof, also suggests is that insomnia may become chronic earlier than is currently outlined in the current diagnostic nosologies (i.e. 3 months). The finding that a previous episode of insomnia was a significant predictor of its persistence, as opposed to remission, was expected as this has been demonstrated in several analytic epidemiological studies examining risk factors for chronic insomnia [40, 41]. What this finding adds is that we should prioritize those with a history of insomnia for treatment during an acute phase. In terms of clinical relevance, the overall findings suggest that interventions aimed to circumvent the transition from acute to chronic insomnia should employ strategies that reduce depression while also addressing sleep-related affective thoughts. While Cognitive Behaviour Therapy for Insomnia (CBT-I) does that by addressing rumination and worry whether a traditional six-eight week face-to-face CBT-I is required is unknown. There is evidence, albeit, preliminary that a briefer version of CBT-I is enough to circumvent this transition [42–44]. The final finding, that higher scores on cognitive and behavioral dimension of sleep preoccupation predicted natural remission was unexpected and deserves consideration. One explanation is that at baseline the individuals with AI had been actively trying these techniques (e.g. going to bed early) or reporting these difficulties (e.g. problems with concentration) but realizing, within the following month, that these strategies offered no relief, stopped, and got better. Examining changes in cognitive and behavioral sleep preoccupation over the course of insomnia would certainly be an interesting next step.

There are limitations within the present study, the most notable of which was the choice of measures and constructs that were used to define predisposing, precipitating, perpetuating, and coping factors. For example, even though fatigue is outlined as one of the nine daytime symptoms of insomnia, according to the ICSD-3, it could equally be argued to be independent of insomnia and not a feature of its perpetuation. While these choices were based upon the previous literature available with regard to AI and subsyndromal insomnia (e.g. [9, 13–15]), they do not account for many other potential variables. Future research may wish to focus on other measurements in each domain, perhaps even including measures of childhood sleep and more information about previous exposure to insomnia and medication history. It could also be argued that including those who had not yet transitioned to chronic insomnia in the final set of analyses (i.e. they were still in the acute phase at follow-up) could limit discussion on the predictors of the transition from pre-chronic to chronic insomnia. While an accurate statement, the small sample size precluded an assessment of those who had transitioned to chronic insomnia (i.e. excluding those who at follow-up still had insomnia but for less than 3 months) compared to those who had remitted. While an examination of the data between baseline and 3-months may have resolved that issue, the drop-out rates between these time points precluded a meaningful analysis. Moreover, whether participants at the second time point were subsyndromal or syndromal is also unknown. While the ISI cut off [33], in addition to the self-reported change in sleep status, from baseline, provide some indication of chronicity, a future study, with increased participant numbers and an increased sampling resolution, may wish to explore these issues further. It could also be argued that the self-report nature of the study is also a limitation. As insomnia is a subjectively defined disorder, however, and there is no conclusive evidence, as yet, of an objective marker of insomnia, it would appear that assessment via self-report is warranted. Finally, the generalizability of the present findings could be questioned. This was a young adult sample, and whereas the demographic make-up of the sample is similar to that seen in the United Kingdom in respect to sex, ethnicity, and marital status [45], this sample contained a higher percentage of educated and full-time students than generally seen in United Kingdom. Future research should replicate the present study with a more diverse population in terms of education and working styles. Moreover, as the age limit was set at 59, the study should be replicated with an older adult sample to determine generalizability within older adults.

In sum, the present study aimed to determine the role of predisposing, precipitating, coping, and perpetuating factors in the development of insomnia. While the findings do suggest that specific elements in each of these domains characterize AI, only anxiety, openness to experience, conscientiousness, insomnia severity affective sleep preoccupation were predictive. Further, only depression scores and levels of sleep preoccupation predicted those whose insomnia would persist from those who will naturally remit. As such, future research should examine the best methods to reduce these symptoms in an effort to prevent chronic insomnia, presumably using a Cognitive Behavioural Therapy for Insomnia framework.

## Funding

This work was funded by the Economic and Social Research Council - Grant No RES-061-25-0120-A.

*Conflict of interest statement.* CAE is a shareholder in Big Health, the company that developed the digital CBT program, Sleepio. He also receives remuneration from Big Health. JGE is director of Sleep Research and Consulting Limited and has served as a consultant for Irish Rugby Football Union, Third City, Mayborn, National Bed Federation, Cohens Veterans Biosciences, Public Health England, and the National Health Service. MAG has served as a consultant for Fitbit, Natrol, Casper Sleep, Merck, Sunovion, Smartypants Vitamins, Pharmavite, and Nightfood. He has received grants from Jazz Pharmaceuticals and Kemin Foods. MG, CHB, and MLP report no financial interests. No authors have any non-financial interests to declare.

## Footnotes

^1^
For the purposes of the report, the term AI covers both the acute and early insomnia phases proposed by Spielman.

^2^
Pilot work demonstrated that individuals with AI would not identify as such and so, following testing, the term “losing sleep due to stress” was used.
